# Belief in COVID-19 Conspiracy Theories, Level of Trust in Government Information, and Willingness to Take COVID-19 Vaccines Among Health Care Workers in Nigeria: Survey Study

**DOI:** 10.2196/41925

**Published:** 2023-05-02

**Authors:** Sunday Oluwafemi Oyeyemi, Stephen Fagbemi, Ismaila Iyanda Busari, Rolf Wynn

**Affiliations:** 1 Department of Community Medicine Faculty of Health Sciences UiT-The Arctic University of Norway Tromsø Norway; 2 Department of Community Health University of Medical Sciences Teaching Hospital Complex Akure, Ondo State Nigeria; 3 Ondo State Ministry of Health Akure Nigeria; 4 Department of Clinical Medicine Faculty of Health Sciences UiT The Arctic University of Norway Tromsø Norway; 5 Department of Education, ICT and Learning Østfold University College Halden Norway

**Keywords:** COVID-19, vaccination, misinformation, conspiracy theories, health workers, Nigeria, government, information, threat, vaccine, willingness, genetic

## Abstract

**Background:**

The World Health Organization recently declared vaccine hesitancy or refusal as a threat to global health. COVID-19 vaccines have been proven efficacious and are central to combatting the pandemic. However, many—including skilled health care workers (HCWs)—have been hesitant in taking the vaccines. Conspiracy theories spread on social media may play a central role in fueling vaccine hesitancy.

**Objective:**

The objective of this study was to investigate HCWs’ belief in COVID-19 vaccine conspiracy theories (ie, that the vaccines can alter one’s DNA or genetic information and that the vaccines contain microchips) and trust in government information on COVID-19 vaccines.

**Methods:**

Health care workers in Ondo State, Nigeria, representing different health care professions were asked to participate anonymously in an online survey. The participants were asked about their beliefs in 2 viral conspiracy theories and their trust in government information on COVID-19 vaccines. We used multivariable logistic regressions to investigate the relationships between trust in government information on COVID-19 vaccines and (1) belief in DNA alteration, (2) belief in microchip implantation through the vaccine, and (3) willingness to accept the vaccine.

**Results:**

A total of 557 HCWs (n=156, 28% men and n=395, 70.9% women) were included in the study. A total of 26.4% (n=147) of the sampled HCWs believed COVID-19 vaccines contained digital microchips, while 30% (n=167) believed the vaccines could alter one’s DNA or genetic information. The beliefs varied according to professional group, with 45.8% (55/120) and 50% (5/10) of nurses and pharmacists, respectively, believing in the DNA alteration theory and 33.3% (40/120) and 37.5% (6/16) of the nurses and laboratory scientists, respectively, believing in the microchip theory. Social media was an important source of COVID-19 information for 45.4% (253/557) of HCWs. A total of 76.2% (419/550) of the participants expressed a willingness to take the vaccine. The odds of HCWs believing that COVID-19 vaccines contained digital microchips increased significantly with decreasing level of trust in government information on COVID-19 vaccines (odds ratio [OR] 4.6, 95% CI 2.6-8.0). We made a similar finding in those who believed COVID-19 vaccines could alter DNA and genetic information (OR 5.2, 95% CI 3.1-8.8).

**Conclusions:**

Misinformation regarding COVID-19 vaccines reaches and influences HCWs. A high proportion of the sampled HCWs believed that COVID-19 vaccines contained microchips or that the vaccines could alter recipients’ DNA and genetic information. This might have negative consequences in terms of the HCWs’ own COVID-19 vaccination and their influence on other people. Lack of trust in government and its institutions might explain the belief in both conspiracy theories and vaccine hesitancy. There is a need for health care stakeholders in Nigeria and around the world to actively counteract misinformation, especially on social media, and give HCWs necessary scientifically sound information.

## Introduction

Vaccination is one of the greatest-ever public health intervention success stories. Globally, vaccination is estimated to avert between 2 and 3 million deaths each year [1]. Vaccination refusal is an impediment to the achievement of successful immunization programs worldwide. The proportion of people refusing to take vaccines may pose a major challenge to the eradication of vaccine-preventable diseases, such as poliomyelitis in Nigeria and India [2-4]. The term “vaccine hesitancy” has been used to describe a continuum between those who accept all vaccines (vaccine acceptance) with no doubts, to those who outright refuse (vaccine refusal) with no doubts. Between these two extremes are the heterogeneous vaccine-hesitant individuals (vaccine hesitancy) [5]. In 2019, the World Health Organization (WHO) listed vaccine hesitancy among the top 10 threats to global health [6]. The justifications for vaccine hesitancy have been described as being complex and context specific and to change over time and place and with the type of vaccine [5,7].

Studies have shown that confidence in vaccination among health care workers (HCWs) has decreased over the past years [8-11]. Vaccine hesitancy or refusal become exceptionally important when those hesitant or refusing to vaccinate are HCWs. This may be a serious concern, because HCWs are still the most trusted counselors and influencers of vaccination decisions [12,13], and their behaviors and opinions could affect the decisions of many who are not HCWs [14]. In addition, unvaccinated HCWs may become infected and transmit the infection to especially vulnerable patients in their care [15].

The COVID-19 vaccines draw on technologies different from those used in traditional vaccines. Even though the messenger RNA (mRNA) vaccine technology is not new, the COVID-19 vaccine by Pfizer/BioNTech was the first mRNA vaccine to go through all the clinical trial stages and obtain approval for emergency use authorization from the US Food and Drug Administration (FDA) on December 11, 2020. The second mRNA vaccine to get similar approval was the Moderna COVID-19 vaccine, on December 18, 2020 [16,17]. Likewise, the viral vector vaccine technology whereby genetic material from the COVID-19 virus is carried by a modified form of another virus (ie, a viral vector), as used by Janssen/Johnson & Johnson (FDA emergency use authorization received on February 27, 2021), AstraZeneca, and the University of Oxford (WHO emergency use authorization received on February 10, 2021) [18], is not new, either [19]. Yet some HCWs have been concerned that the COVID-19 vaccines could alter their DNA or genetic information. Recently, after the completion of this survey, the Pfizer/BioNTech and Moderna mRNA vaccines gained full FDA approval, while the Janssen/Johnson & Johnson vaccine received restricted approval [20].

Reports from different parts of the world, including Europe, the United States, and Africa, have suggested that many HCWs have been hesitant to be vaccinated against COVID-19 [15,21,22]. The COVID-19 vaccine has been tangled in perceived vaccine risks, fueled by misconceptions, rumors, spurious controversies, implausible conspiracy theories, and misinformation, especially on social media [23-25]. Health-related misinformation on social media is an increasing concern, as this misinformation has been shown to spread rapidly and to impact health behavior in a range of areas, including attitudes to vaccination [26,27]. A range of conspiracy theories relating to the novel COVID-19 vaccines have circulated on social media [28]. One of the popular conspiracy theories at the time of the study involved the idea that the vaccines contained microchips that could be used, for instance, to track those vaccinated. Another involved the idea that the vaccines could damage the DNA of those who received them. A common denominator for these and other conspiracy theories relating to the COVID-19 vaccines is a lack of trust in the government’s information on COVID-19 and the vaccines.

This study aims to investigate HCWs’ belief in COVID-19 conspiracy theories (ie, that the vaccines can alter one’s DNA or genetic information and that the vaccines contain digital microchips) and associations with the level of trust in government information and willingness to take the COVID-19 vaccines. This is intended to provide deeper insight into the factors that may be responsible for the low uptake of the COVID-19 vaccines by HCWs in Africa, as reported by the WHO [29,30], and issues that may need to be addressed to reduce vaccine hesitancy or outright refusal among HCWs.

## Methods

### Ethics Approval

The study was anonymous, and the informed consent of the participants was sought before they filled in the questionnaire. No compensation or incentives were given to the participants. The study was approved by the Health Research Ethics Committee of the Federal Medical Centre at Owo in Ondo State, Nigeria (FMC/OW/380/VOL.CX/74).

### Study Population

The study was performed in Ondo State, southwest Nigeria. Professional groups for HCWs in this questionnaire-based study included medical doctors, nurses, pharmacists, laboratory scientists, community health extension officers or workers, health assistants, and others. All respondents worked in Ondo State, southwest Nigeria. The questionnaire was open for about two months, from March 9, 2021, to May 11, 2021. A total of 564 participants answered the questionnaire. We excluded those who opened or started the questionnaire but answered less than one-tenth of the questions. The final study sample consisted of 557 participants.

### Recruitment

First, we informed the leaders and some members of each HCW professional group about the study. A text message (via SMS) drafted by the authors was then posted by the leader or members of the group on the groups’ WhatsApp platforms on mobile phones. The SMS carried a link to the online questionnaire, where interested HCWs could voluntarily participate in the study. The online questionnaire was designed and conducted using Nettskjema [31]. The WhatsApp mobile app is the most popular means of disseminating information to HCWs in Ondo State, where all HCW professional groups have group WhatsApp platforms.

### Questionnaire Items

The participants were asked to give some basic demographic information and information about their work: their gender (man or woman), age group (<20, 20-29, 30-39, 40-49, 50-59, and >59 years), marital status (single, married, divorced, or widowed), level of education (secondary, university, postgraduate, or other), type of HCW (medical doctor, nurse, pharmacist, laboratory scientist, community health extension officer, health assistant, or other), and the type of health care facility where they worked (primary, secondary, or tertiary health facility; private hospital; or other). They were also asked to give information about any chronic illness (“yes,” “no,” or “I don’t know”) and prior COVID-19 infection (“yes,” “no,” “I believe I had the infection even though I did not do a test to confirm it,” or “I don’t know”).

The participants were asked about their trust in government information on COVID-19 and vaccines with the following question: “On a scale of 10 where 1= least trusted and 10=most trusted, how much do you trust the government regarding COVID-19 information and vaccine?”

The HCWs were requested to respond to 2 questions relating to highly circulated conspiracy theories: “I think COVID-19 vaccine is a means to implant digital microchips to track and control people” and “I think COVID-19 vaccine will alter my DNA or genetic information.” Responses to each of the statements included “strongly agree,” “agree,” “neutral,” “disagree,” or “strongly disagree.” The participants were also asked the following question: “If COVID-19 vaccine is available and free, will you take it?” The participants chose a response option from the following: “yes,” “no,” “I don’t know,” and “I will intentionally delay for months.” If participants were not willing to take the vaccine, they were asked why (possible reasons included “safety concern,” “lack of trust in the government,” “scared of COVID-19 vaccine,” “vaccine is against my religion,” and other reasons). They were further asked to choose their main trusted source or sources of information on COVID-19 and vaccines from the following: social media (ie, WhatsApp and Facebook), traditional media (ie, TV and newspaper), health authorities (state and federal), colleagues and friends, academic journals, and others.

### Analytical Variables

We categorized responses to the question “How much do you trust the government regarding COVID-19 information and vaccine?” as 1 to 4 (low level of trust), 5 to 6 (medium level of trust), and 7 to 10 (high level of trust). We dichotomized the response to the statement “COVID-19 vaccine is a means to implant digital microchips to track and control people” by coalescing “strongly agree,” “agree,” and “neutral” into “yes” and “strongly disagree” and “disagree” into “no.” Likewise, we dichotomized “COVID-19 vaccine will alter my DNA or genetic information” by coalescing “strongly agree,” “agree,” and “neutral” into “yes” and “strongly disagree” and “disagree” into “no.” Lastly, we dichotomized the response to the question “If COVID-19 vaccine is available and free, will you take it?” into “yes” as “vaccine acceptance” and coalescing “no,” “I don’t know,” and “I will intentionally delay for months” into “vaccine hesitancy.” We used the resulting variable for the third logistic regression.

### Statistical Methods

We used descriptive statistics to calculate and summarize all the variables as absolute values and percentages. We used multivariable logistic models to fit the following three dichotomized outcomes: (1) “COVID-19 vaccine is a means to implant digital microchips into people,” (2) “COVID-19 vaccine will alter my DNA or genetic information,” and (3) “If COVID-19 vaccine is available and free, will you take it.” These were used as the dependent variables in each of the 3 main logistic regressions. We used purposeful selection of independent variables for each of the 3 main logistic regression models [32]. Any independent variable selected for any of the 3 main models using this method was also used in all the models, that is, the same independent variables were used in all models.

All statistical analyses were conducted using Stata (version 17.0; Stata Corp). All *P* values were considered statistically significant at a level of <.05.

## Results

The demographic characteristics of the participating HCWs and the missing values are summarized in Tables 1 and 2. Of the 557 participants, 395 (71.7%) were women and 156 (28.3%) were men. The mean age group of both men and women was 40 to 49 years, with about 80.6% (444/551) having a university or postgraduate degree; 12.5% (69/552) were medical doctors, 21.7% (120/552) were nurses, and 41.7% (230/552) were community health extension officers. The pharmacists, laboratory scientists, and health assistants combined were less than 5% of the study population (Table 1).

**Table 1:** Overall respondents and missing values in the survey (n=557).

Variable		Respondents, n (%)	Missing values, n (%)	
**Gender**				6 (1.1)
	Women	395 (70.9)		
	Men	156 (28)		
**Age group (years)**				3 (0.5)
	20-29	36 (6.5)		
	30-39	119 (21.4)		
	40-49	229 (41.1)		
	50-59	156 (28)		
	>59	14 (2.5)		
**Marital status**				4 (0.7)
	Single	50 (9)		
	Married	490 (88)		
	Divorced	4 (0.7)		
	Widowed	9 (1.6)		
**Health care worker category**				5 (0.9)
	Medical doctor	69 (12.4)		
	Nurse	120 (21.5)		
	Pharmacist	10 (1.8)		
	Laboratory scientist	16 (2.9)		
	Community health extension officer	230 (41.3)		
	Health assistant	4 (0.7)		
	Other	103 (18.5)		
**Education**				6 (1.1)
	Secondary	4 (0.7)		
	University degree	289 (51.9)		
	Postgraduate	155 (27.8)		
	Other	103 (18.5)		
**Chronic illness**				3 (0.5)
	No	53 (9.5)		
	Yes	496 (89.1)		
	I don’t know	5 (0.9)		
**Main and trusted information sources on COVID-19 and vaccines**				0 (0)
	Social media (WhatsApp, Facebook)	253 (45.4)		
	Traditional media (TV, newspaper)	153 (27.5)		
	Health authorities (federal, state)	459 (82.4)		
	Colleagues/friends	99 (17.8)		
	Academic journal	113 (20.3)		
	Other	30 (5.4)		
**Response to “I think COVID-19 vaccine contains digital microchips”**				7 (1.3)
	No	403 (72.3)		
	Yes	147 (26.4)		
**Response to “I think COVID-19 vaccine will alter my DNA and genetic information”**				10 (1.8)
	No	380 (68.2)		
	Yes	167 (30)		
**Response to “Will you take COVID-19 vaccine?”**				7 (1.3)
	Vaccine hesitance	131 (23.5)		
	Vaccine acceptance	419 (75.2)		
**Level of trust in government information on COVID-19**				16 (2.9)
	Low level of trust (score 1-4)	199 (35.7)		
	Medium level of trust (score 5-6)	96 (17.2)		
	High level of trust (score 7-10)	246 (44.2)		

**Table 2:** Descriptive characteristics of the study population (n=557). The sum of absolute values may not add up to 557 because of different missing values in different variables (the precise number of respondents in each category and missing values can be found in Table 1).

		“I think COVID-19 vaccine contains digital microchips,” respondents (n=550), n (%)			“I think COVID-19 vaccine will alter my DNA and genetic information,” respondents (n=547), n (%)			“Will you take COVID-19 vaccine?” respondents (n=550), n (%)	
		Yes	No	Yes		No	Vaccine acceptance		Vaccine hesitance
Overall		147 (26.7)	403 (73.3)	167 (30.5)		380 (69.5)	419 (76.2)		131 (23.8)
**Level of trust in government information**									
	Low (score 1-4)	81 (40.9)	117 (59.1)	100 (50.5)		98 (49.5)	94 (47.2)		105 (52.8)
	Medium (score 5-6)	28 (29.5)	67 (70.5)	30 (31.6)		65 (68.4)	80 (85.1)		14 (14.9)
	High (score 7-10)	35 (14.5)	207 (85.5)	36 (14.9)		206 (85.1)	229 (95)		12 (5)
**Gender**									
	Women	107 (27.3)	285 (72.7)	108 (27.8)		280 (72.2)	300 (77.1)		89 (22.9)
	Men	38 (25)	114 (75)	56 (36.6)		97 (63.4)	114 (73.6)		41 (26.4)
**Age group (in years)**									
	20-29	14 (38.9)	22 (61.1)	10 (27.8)		26 (72.2)	25 (69.4)		11 (30.6)
	30-39	34 (29.1)	83 (70.9)	46 (39.7)		70 (60.3)	82 (69.5)		36 (30.5)
	40-49	59 (26)	168 (74)	59 (26.2)		166 (73.8)	176 (78.6)		48 (21.4)
	50-59	34 (21.9)	121 (78.1)	47 (30.7)		106 (69.3)	121 (78.1)		34 (21.9)
	>59	5 (38.5)	8 (61.5)	4 (28.6)		10 (71.4)	12 (85.7)		2 (14.3)
**Marital status**									
	Single	22 (44)	28 (56)	16 (32)		34 (68)	43 (86)		7 (14)
	Married	118 (24.4)	365 (75.6)	141 (29.4)		339 (70.6)	365 (75.6)		118 (24.4)
	Divorced	2 (50)	2 (50)	2 (50)		2 (50)	2 (50)		2 (50)
	Widowed	3 (33.3)	6 (66.7)	5 (55.6)		4 (44.4)	7 (77.8)		2 (22.2)
**Categories of health care worker**									
	Medical doctor	8 (11.8)	60 (88.2)	21 (31.3)		46 (68.7)	50 (72.5)		19 (27.5)
	Nurse	40 (33.3)	80 (66.7)	55 (45.8)		65 (54.29	59 (50)		59 (50)
	Pharmacist	2 (20)	8 (80)	5 (50)		5 (50)	8 (80)		2 (20)
	Laboratory scientist	6 (37.5)	10 (62.5)	6 (37.5)		10 (62.5)	12 (80)		3 (20)
	Community health extension officer	55 (24.3)	171 (75.7)	43 (19.3)		180 (80.7)	205 (90.3)		22 (9.7)
	Health assistant	0 (0)	4 (100)	1 (25)		3 (75)	3 (100)		0 (0)
	Other	35 (34.3)	67 (65.7)	35 (34.3)		67 (65.7)	77 (74.8)		26 (25.2)
**Education**									
	Secondary	0 (0)	4 (100)	1 (25)		3 (75)	4 (100)		0 (0)
	University degree	75 (26.2)	210 (73.7)	93 (32.6)		192 (67.4)	214 (74.6)		73 (25.4)
	Postgraduate	36 (23.4)	118 (76.6)	47 (30.7)		106 (69.3)	113 (73.9)		40 (26.1)
	Other	34 (33.7)	67 (66.3)	25 (25.2)		74 (74.8)	82 (82)		18 (18)
**Chronic illness**									
	Yes	15 (28.8)	37 (71.2)	24 (47.1)		27 (52.9)	35 (67.3)		17 (32.7)
	No	130 (26.5)	360 (73.5)	139 (28.5)		349 (71.5)	379 (77.4)		111 (22.6)

Interestingly, beliefs in the 2 vaccine-related conspiracy theories were high among the responding health workers. As many as 26.7% (147/550) of the respondents did not reject the idea that COVID-19 vaccines contained microchips that could be used for surveillance, and 30.5% (167/547) thought that the vaccines could alter one’s DNA or genetic information.

Beliefs also varied according to profession, with 45.8% and 50% of nurses and pharmacists, respectively, believing in the DNA/genetic information alteration theory and 33.3% and 37.5% of nurses and laboratory scientists, respectively, believing in the microchip theory. Social media was an important source of COVID-19 information for 45.4% of HCWs (Table 1).

There were some gender-related differences. A higher proportion of women (107/392, 27.3%) thought that the COVID-19 vaccines contained digital microchips compared to men (38/152, 25%), while a higher proportion of men (56/153, 36.6%) thought that the COVID-19 vaccines would alter their DNA compared to women (108/388, 27.8%). In spite of the aforesaid differences, a higher proportion of women (300/389, 77.1%) were willing to accept the vaccine compared to men (114/155, 73.6%; Table 2).

Trust in COVID-19 information from the government was related to vaccine acceptance, as a higher proportion (229/241, 95%) of those who had a high level of trust in the government’s information on COVID-19 and the vaccine were willing to accept the vaccine compared to those with a low level of trust (94/199, 47.2%; Table 2).

Trust in the government’s information was related to belief in the conspiracy theories and to vaccine acceptance. The odds of not rejecting the conspiracy theory that the COVID-19 vaccines contain digital microchips increased significantly with a decreasing level of trust in the government’s information regarding COVID-19 and the vaccines (odds ratio [OR] 4.6, 95% CI 2.6-8.0) when compared to those with a high level of trust. Findings were similar in those who did not reject the conspiracy theory that the COVID-19 vaccine would alter their DNA (OR 5.2, 95% CI 3.1-8.8). The findings remained significant after adjusting for multiple covariates (Table 3). Likewise, the odds of COVID-19 vaccine acceptance increased significantly with increasing level of trust in the government’s information on COVID-19 and the vaccine (OR 18.5 95% CI 8.8-39.1) when compared to a low level of trust. This finding remained significant after adjusting for multiple covariates (Table 3).

Those who obtained their main COVID-19 information from the health authority had increased odds of taking the COVID-19 vaccine (OR 2.1 95% CI 1.0-4.2) compared to those who did not (Table 4).

**Table 3:** Odds ratios with the 95% CI for belief in microchips in COVID-19 vaccines, DNA-altering vaccines, and willingness to take COVID-19 vaccines by level of trust in government information on COVID-19 and vaccines. Multivariable 1 adjusted for age, sex, marital status, education level, category of health care work, place of work, and chronic illness; multivariable 2 adjusted for the above variables in addition to prior COVID-19 infection, daily exposure to COVID-19, and main and trusted source or sources of COVID-19 information.

Models		Low trust (score 1-4), OR^a^ (95% CI)	Medium trust (score 5-6), OR (95% CI)	High trust (score 9-10), OR (95% CI)	*P* value
“**I think COVID-19 vaccine contains digital microchips”**					
	Unadjusted	4.1 (2.6-6.5)	2.5 (1.4-4.4)	1.0	<.001
	Multivariable 1	4.7 (2.7-8.0)	2.9 (1.5-5.4)	1.0	<.001
	Multivariable 2	4.4 (2.5-7.7)	2.8 (1.4-5.5)	1.0	<.001
“**I think COVID-19 vaccine will alter my DNA and genetic information”**					
	Unadjusted	5.8 (3.7-9.2)	2.6 (1.5-4.6)	1.0	<.001
	Multivariable 1	5.4 (3.2-9.1)	2.8 (1.5-5.1)	1.0	<.001
	Multivariable 2	5.2 (3.0-8.9)	2.5 (1.3-4.8)	1.0	<.001
“**Will I take COVID-19 vaccine?”**					
	Unadjusted	1.0	6.4 (3.4-12.0)	21.3 (11.2-40.6)	<.001
	Multivariable 1	1.0	6.8 (3.4-13.9)	20.6 (9.8-43.2)	<.001
	Multivariable 2	1.0	8.2 (3.8-17.4)	18.5 (8.7-39.4)	<.001

^a^OR: odds ratio.

**Table 4:** Adjusted odds ratios (with 95% CI) of comparisons within gender, age groups, and health care worker categories regarding belief in microchips in the COVID-19 vaccine, DNA-altering vaccines, and willingness to take the COVID-19 vaccines. The models were adjusted for age, sex, marital status, education level, health care worker category, place of work, chronic illness, prior COVID-19 infection, daily exposure to COVID-19, main and trusted source or sources of COVID-19 information, and level of trust in government information on COVID-19.

		“I think the COVID-19 vaccine contains digital microchips,” aOR^a^ (95% CI)	“I think the COVID-19 vaccine will alter my DNA and genetic information,” aOR (95% CI)	“I will take the COVID-19 vaccine,” aOR (95% CI)
**Gender**				
	Women	1.0	1.0	1.0
	Men	1.4 (0.8-2.5)	1.8 (1.1-3.2)	1.0 (0.5-2.0)
**Age group (years)**				
	20-29	1.0	1.0	1.0
	30-39	1.5 (0.5-4.3)	2.4 (0.8-7.0)	2.5 (0.6-9.9)
	40-49	2.0 (0.7-5.8)	1.5 (0.5-4.7)	2.1 (0.5-8.4)
	50-59	1.7 (0.6-5.2)	2.1 (0.7-6.7)	2.5 (0.6-10.3)
	>59	2.9 (0.4-18.2)	1.9 (0.3-12.5)	3.1 (0.3-35.9)
**Categories of health care worker**				
	Medical doctors	1.0	1.0	1.0
	Nurses	3.9 (1.3-12.0)	2.2 (0.9-5.4)	0.5 (0.2-1.4)
	Pharmacists	3.0 (0.4-22.0)	3.1 (0.6-16.2)	2.1 (0.3-14.9)
	Laboratory scientists	5.1 (1.0-25.9)	1.9 (0.4-7.9)	1.5 (0.2-8.6)
	Community health extension officers	4.0 (1.2-13.8)	1.7 (0.6-4.5)	2.2 (0.7-7.5)
	Health assistants	N/A^b^	2.7 (0.1-162.0)	N/A
	Others	10.5 (3.1-35.7)	2.8 (1.1-7.3)	1.0 (0.3-3.1)
**Health authority as the main source of COVID-19 information**				
	No	1.0	1.0	1.0
	Yes	0.4 (0.2-0.7)	0.5 (0.3-0.9)	2.1 (1.0-4.2)

^a^aOR: adjusted odd ratio.

^b^N/A: not applicable.

## Discussion

### Principal Results

One main finding in this study is the relatively high proportion of health workers who believed that the vaccines contained microchips and that the vaccines could alter the recipients’ DNA or genetic information. More than a quarter of the respondents believed in 1 of these 2 conspiracy theories. We lack information about why so many of the HCWs believed in these misconceptions about the COVID-19 vaccines. Even though technologies that are different from older, long-established ones were used to develop the different COVID-19 vaccines, such as the mRNA vaccine technology utilized by Pfizer/BioNTech and Moderna [16] and the viral vector vaccine technology utilized by Janssen/Johnson & Johnson, AstraZeneca, and the University of Oxford [19], there are still no compelling justifications for HCWs to believe the misleading vaccine conspiracy theories. However, there are historical reasons, including alleged unethical practices by pharmaceutical companies in Nigeria, that may play a role in fueling skepticism toward vaccines in the country [33].

The central importance of trust has been underlined in studies focusing on vaccine hesitancy [34]. A lack of trust in the government and its institutions has also been highlighted as an important reason underlying vaccine hesitancy in Nigeria [35]. In our study, a higher level of trust in government information on COVID-19 vaccines was positively related to a lower risk of belief in conspiracy theories and an increase in COVID-19 vaccine acceptance and could therefore be an underlying explanatory factor. Simply put, not trusting the government may increase the likelihood of believing in conspiracy theories and decrease the acceptance of vaccines among HCWs.

Nigeria plays a central role in democracy in Africa and is the most populous country on the continent. However, the country has recently witnessed political unrest, violence, and terrorism. Mistrust in government could be related to a lack of government accountability in public-sector management [36], and there is a need to further strengthen security and governance to improve the population’s trust in its governing institutions [37].

More women believed in the microchip conspiracy and more men in the DNA conspiracy, but the differences in beliefs between the genders were only statistically significant for the latter (Table 4), suggesting that the respondents’ beliefs in conspiracies may occasionally be gender specific. There were also some contrasts between the different HCW groups, with the nurses and the pharmacists having the most respondents with conspiracy theory beliefs; as many as 45.8% (55/120) and 50% (5/10), respectively, believed that the vaccines could alter their DNA. The nurses and the laboratory scientists were the occupational groups with the highest proportion of believers (40/120, 33.3%, and 6/16, 37.5%, respectively) in the microchip conspiracy. When calculating adjusted ORs for belief in the conspiracy theories (Table 4), the nurses, the community health extension officers, and the “other” category stood out as having significantly higher levels of conspiracy theory beliefs. There were no clear patterns regarding the importance of age or marital status for belief in the 2 conspiracy theories.

Approximately three-quarters of the respondents were willing to take the COVID-19 vaccine, a higher proportion than previously reported among HCWs in northern Nigeria [38]. The level of trust in government was clearly related to an increased willingness to take the vaccine (Table 4). A prior study [39] found an increased willingness to take the vaccine in older respondents, and a similar but nonsignificant trend was found in this study [39]. In contrast to prior findings, the well-established risk factor for a more severe infection of having a chronic illness was not related to an increased willingness to take the vaccine [39]. However, no subcategorization was made in our study regarding type of chronic illness, and only some chronic illnesses are related to a worsened COVID-19 outcome. A lower willingness has been noted in particular among nurses in prior studies [26,38], and in our study, only half of the nurses stated that they were willing to take the vaccine, while this was the case with about three-quarters or more in the other professional groups. However, when calculating adjusted ORs (Table 4), the willingness to take the vaccine was not significantly different between the different professional groups. We lack data regarding why nurses seem to be the professional group with the highest belief in conspiracies, but it may be related to the contrasting groups, including the medical doctors, having more knowledge regarding vaccine technologies.

Probably unsurprisingly, a large proportion of these HCWs (253/557, 45.4%) obtained their trusted information about COVID-19 from social media—where we know much health-related misinformation is propagated [26,27]. In our study, those who stated they relied on government institutions as their main source of COVID-19–related information were significantly less likely to believe in the conspiracy theories (Table 4). Prior studies have also found that many HCWs rely on social media for COVID-19–related information [40]. A recent study found that there is an increasing belief in Nigeria that vaccines are unsafe [41]. This is likely in part fueled by social media misinformation [23,26,27].

We believe our study underlines the need to actively counteract misleading and false information on social media and to make sure that HCWs are given scientifically sound information on this important topic. This information should be given by different stakeholders (eg, the government and nongovernmental organizations) through different channels, including social media, in order to reach as many HCWs as possible. HCWs are clearly not immune to social media misinformation.

### Limitations

This study has some limitations that one should be aware of while interpreting the results. The study used the WhatsApp platforms of the various professional groups to distribute the questionnaire. Any member who was not on the platform or was not connected to the internet during the period of data collection would potentially not have been reached by our questionnaire. However, most members are usually on the professional groups’ WhatsApp platforms because this is where they obtain up-to-date group information and communicate among themselves across health facilities, cities, and towns. We lack detailed information regarding the number of people that were members of the different professional groups and received the invitation to participate; we are therefore not able to calculate an accurate response rate. However, studies with online recruitment typically achieve relatively low response rates, and this is likely to have been the case and a limitation in this study [42]. With our method of data collection, it was possible for a member of a group to answer the questionnaire multiple times. However, we do not see any reason why an individual would do so. The study was carried out in Ondo State, one of Nigeria’s 36 states. Keeping the limitations mentioned above in mind, we believe the study’s findings may be representative of HCWs in this state, as well as of neighboring southwestern states, which in many ways are relatively similar. It is more uncertain to what extent the study’s findings can be generalized to all of Nigeria, which is the most populous country in Africa and quite diverse in terms of ethnic groups, languages, and cultures.

### Conclusions

A relatively high proportion of the HCWs expressed belief in the microchip or DNA conspiracy theories, or both, and only about three-quarters were willing to take the COVID-19 vaccine. A lack of trust in the government’s information and its institutions might explain both the belief in the conspiracies as well as the vaccine hesitancy. Health workers are not immune to misinformation, and social media plays an important part in fueling vaccine hesitancy. There is a need for stakeholders to actively counteract social media misinformation and to provide health workers with scientifically sound information.

## Abbreviations

HCW: health care worker
mRNA: messenger RNA
OR: odds ratio
WHO: World Health Organization

## Data Availability

The data set generated and analyzed during the current study are available from the corresponding author on reasonable request.
